# Paternal kin recognition in the high frequency / ultrasonic range in a solitary foraging mammal

**DOI:** 10.1186/1472-6785-12-26

**Published:** 2012-11-30

**Authors:** Sharon E Kessler, Marina Scheumann, Leanne T Nash, Elke Zimmermann

**Affiliations:** 1 School of Human Evolution & Social Change (SHESC), Arizona State University, Box 872402, Tempe, AZ, 85287-2402, USA; 2 Institute of Zoology, University of Veterinary Medicine Hannover, Buenteweg 17, Hannover, 30559, Germany

**Keywords:** Kin selection, Inbreeding avoidance, Social complexity, Vocalization

## Abstract

**Background:**

Kin selection is a driving force in the evolution of mammalian social complexity. Recognition of paternal kin using vocalizations occurs in taxa with cohesive, complex social groups. This is the first investigation of paternal kin recognition via vocalizations in a small-brained, solitary foraging mammal, the grey mouse lemur *(Microcebus murinus*), a frequent model for ancestral primates. We analyzed the high frequency/ultrasonic male advertisement (courtship) call and alarm call.

**Results:**

Multi-parametric analyses of the calls’ acoustic parameters and discriminant function analyses showed that advertisement calls, but not alarm calls, contain patrilineal signatures. Playback experiments controlling for familiarity showed that females paid more attention to advertisement calls from unrelated males than from their fathers. Reactions to alarm calls from unrelated males and fathers did not differ.

**Conclusions:**

1) Findings provide the first evidence of paternal kin recognition via vocalizations in a small-brained, solitarily foraging mammal. 2) High predation, small body size, and dispersed social systems may select for acoustic paternal kin recognition in the high frequency/ultrasonic ranges, thus limiting risks of inbreeding and eavesdropping by predators or conspecific competitors. 3) Paternal kin recognition via vocalizations in mammals is not dependent upon a large brain and high social complexity, but may already have been an integral part of the dispersed social networks from which more complex, kin-based sociality emerged.

## Background

Though kin selection (the preferential treatment of genetic relatives) has been theorized to be one of the most important forces driving the evolution of social complexity in mammals, we still know surprisingly little about how this process occurs [1,2]. Vocalizations are an important cue for the recognition of maternal kin (related through the mother) in species with large brains, complex social systems and cohesive foraging groups (primates [3-5], hyenas [6], elephants [7,8], dolphins [9], pinnipeds [10], [11-13]) and in small-brained species with varying degrees of social complexity (colony-living bats: [14,15], small-brained, group-living lemurs [16], and the socially variable house mouse (full-sibling recognition: [17-19])). Far less is known about recognition of paternal kin (related through the father), though it is expected to shape the evolution of social behavior through paternal kin selection and inbreeding avoidance [1,2,20]. Long-term field studies of species with complex social systems suggest they often behave as if they recognize paternal kin (baboons: [21], hyenas: [22,23], reviews: [20,24]). Studies investigating the cues have shown that large-brained macaques use vocalizations for paternal kin recognition [25,26] and that small-brained laboratory rodents use olfaction (i.e., [27,28], review: [20]). To our knowledge, our study is the first to demonstrate acoustic patrilineal signatures and paternal kin recognition via vocalizations in a solitary-foraging mammal, suggesting that this ability can evolve independently of social complexity.

We investigated the grey mouse lemur (*Microcebus murinus*) as a model for small-brained mammals with relatively simple social systems [13,29]. Within primates, it retains basal morphological traits including a small brain-size relative to body size [13] and has been suggested to represent an ancestral primate model [29,30]. It is a tiny, nocturnal strepsirrhine primate endemic to Madagascar that maintains social networks involving shared home ranges and sleeping sites, but forages alone for insects and fruit in thin, terminal ends of tree branches in tropical forests [31-34]. This is a particularly interesting species in which to investigate paternal kin recognition via vocalizations, because in the wild females are philopatric and cooperatively raise their young in nests with maternal kin [31,33]. Males provide no paternal care and do not co-nest with their mates or with their young, thus limiting the opportunities for the familiarity-based mechanisms seen in species with more complex social systems ([20,31,33-36]). However, inbreeding avoidance is still likely to be highly important, because males may remain in the same area for multiple years and during the breeding season they can expand their ranges to be more than twice as large as the females’ ranges, making it likely that adult males’ ranges will overlap the ranges of their daughters from previous mating seasons [32,37].

Because mouse lemurs are nocturnal, solitary-foragers living in dense forests, vocal communication is highly important for regulating social interactions across distances where visibility is poor and olfactory communication is limited [38]. Mouse lemurs suffer from high predation [39], and their high frequency and ultrasonic calls have been suggested to be an anti-predator strategy by calling above the hearing range of owls [38]. Two of the most frequent calls are the mate advertisement call and the alarm call. The mate advertisement call is used in social and sexual contexts [40,41]. It is a complex, high frequency / ultrasonic vocalization that starts with a whistle unit, followed by an upward sweep, and a highly modulated tail unit [40,41]. The alarm call is given in social and disturbance contexts and it is a short, almost non-modulated, high frequency call [40]. Both call types contain individual signatures [40,41]. If used for paternal kin recognition, both call types could facilitate kin selection, and the advertisement call could also enable inbreeding avoidance in sexual contexts.

We tested two hypotheses in each call type: (i) *Patriline Signature Hypothesis:* calls will be distinctive by patriline, and (ii) *Patriline Recognition Hypothesis:* females will respond differently to calls from their fathers and unrelated males when familiarity is controlled. We found patrilineal signatures and paternal kin recognition in the high frequency/ultrasonic male advertisement call but not in the high frequency alarm call. These findings suggest that paternal kin recognition via vocalizations can emerge in mammals independently of a large brain and high level of social complexity.

## Results

### Patriline signatures

**Figure 1:**
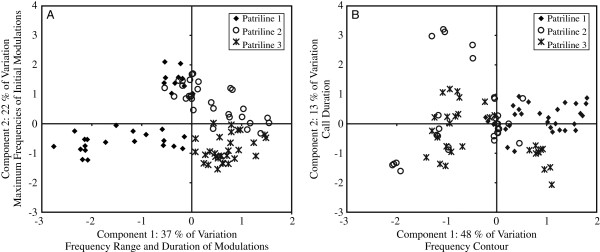
**Separation of calls by patriline produced by components 1 and 2.A**: advertisement calls, **B**: alarm calls. Only the advertisement calls showed statistically significant classification by patriline. See Table 1 and Additional file 1 for the quartiles of each acoustic parameter and their loadings on the components.

Advertisement calls, but not alarm calls, contained patrilineal signatures. Seventy nine percent of the advertisement calls and 45% of the alarm calls were correctly assigned to their respective patrilines (permutated discriminant function analysis, *chance* = 33*%*, *P*_*adverstisement* _ *call*_ = 0.0398, *P*_*alarm* _ *call*_ = 0.609). Figure 1 shows the separation of advertisement calls and alarm calls by patriline produced by the principal components analyses (see also Table 1 and Additional file 1, which summarize the data). Because the acoustic structures of the calls are complex, principal components analysis was used to reduce the number of parameters [42]. For the advertisement calls, high positive values on component 1 (37% of the variation) were associated with modulations of a longer duration and a greater frequency range and higher maximum frequencies in the tail modulations. High positive values on component 2 (22% of the variation) are associated with high maximum frequencies in the first seven modulations.

**Table 1:** Quartiles of the acoustic parameters measured from the advertisement calls and their loadings on the principal components

**Acoustic parameter**	**Quartiles**			**Component loadings**	
	**25**	**50**	**75**	**Component 1**	**Component 2**
**Frequency range of modulation six**	11428.00	13143.00	14286.00	.858	.096
**Frequency range of modulation five**	11066.00	12653.00	14286.00	.852	-.053
**Max**. **frequency of third modulation from end**	19518.50	21633.00	23306.00	.845	-.266
**Max**. **frequency of the modulation before the end**	15918.00	17714.00	20000.00	.843	-.373
**Max**. **frequency of the second modulation from the end**	17193.00	19429.00	21857.00	.835	-.371
**Frequency range of modulation four**	10235.50	12000.00	13714.25	.823	-.151
**Frequency range of modulation seven**	11228.00	13265.50	14694.00	.800	.197
**Max**. **frequency of end modulation**	14286.00	15510.00	16612.00	.799	-.246
**Frequency range of the third modulation from the end**	5714.75	7975.00	10428.00	.762	-.438
**Fundamental frequency of the end**	11719.00	13086.00	14697.00	.750	-.093
**Frequency range of the third modulation**	8421.00	10857.00	13143.00	.733	-.372
**Duration of third modulation from the end**	8.00	10.00	13.00	.718	-.037
**Frequency range of the second modulation from the end**	4543.25	6216.50	9316.50	.711	-.551
**Fundamental frequency of the start**	20325.00	23499.00	24853.75	.704	.572
**Duration of the modulation before the end**	7.00	9.00	13.00	.687	-.211
**Duration of the second modulation before the end**	7.75	10.00	12.00	.682	-.177
**Frequency range of the modulation before the end**	3265.75	4905.00	8164.00	.676	-.575
**Duration of modulation four**	11.00	13.00	14.00	.669	-.153
**Duration of the end modulation**	5.00	10.00	13.00	.652	-.452
**Duration of modulation six**	12.00	13.00	15.00	.620	-.031
**Number of modulations**	18.00	20.00	23.00	-.620	.057
**Frequency range of the end modulation**	2844.50	3844.00	6129.00	.613	-.584
**Duration of modulation five**	11.75	13.00	14.25	.596	-.148
**Time until the turning point**	36.00	42.00	53.25	-.561	.021
**Frequency range of modulation two**	6939.00	8496.00	11275.75	.553	-.513
**Duration of modulation seven**	12.00	13.00	15.00	.541	.094
**Duration of modulation three**	11.00	12.00	13.00	.536	-.074
**Duration of modulation two**	10.00	11.50	13.00	.519	-.440
**Time until the call**'**s maximum**	68.75	78.50	89.25	-.434	-.127
**Call duration**	594.75	656.50	734.75	-.406	-.133
**Duration of modulation one**	8.00	10.00	11.00	.252	-.070
**Peak frequency of the end**	12219.50	13513.50	15997.25	.250	.107
**Max**. **frequency of modulation three**	27551.00	31143.00	33917.25	.399	.886
**Max**. **frequency of modulation two**	29478.00	32571.00	35714.00	.407	.868
**Max**. **frequency of modulation four**	26639.00	29959.00	32245.00	.481	.835
**Max**. **frequency of modulation five**	26286.00	29714.00	31020.00	.548	.779
**Fundamental frequency of the turning point**	21851.00	24078.50	26739.25	.345	.756
**Max**. **frequency of modulation one**	31358.75	34286.00	37143.00	.546	.752
**Max**. **frequency of modulation six**	26046.50	28775.50	30367.50	.583	.746
**Max**. **frequency of modulation seven**	25410.00	28367.00	29592.00	.574	.739
**Fundamental frequency of the maximum**	27466.00	31372.00	34081.75	.505	.737
**Peak frequency of the maximum**	27881.00	31787.00	34668.00	.128	.622
**Peak frequency of the turning point**	21851.00	24373.50	27197.00	.281	.561
**Peak frequency of the start**	20508.00	24292.00	28284.00	.120	.458
**Frequency range of modulation one**	4571.00	5714.00	7194.00	.301	-.391

Frequency is measured in Herz and time in milliseconds. Components 1 and 2 are 37% and 22% of the variation, respectively.

Acoustic dissimilarity between dyads correlated significantly with patrilineal genetic dissimilarity between dyads (Mantel test: r=0.191, g=1.9327, Z=6.5104, p=0.028) and did not correlate with matrilineal genetic dissimilarity between dyads (Mantel test: r=−0.0721, g=−0.3679, Z=7.1612, p=0.4120).

### Patriline recognition

**Figure 2:**
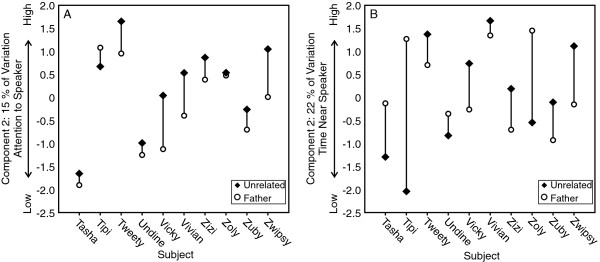
**Females**’ **responses to advertisement calls** (**A**) **and alarm calls** (**B**) **from their fathers and unrelated males.A**: Component 2 showed that nine of 10 females paid more attention to the advertisement calls of the unrelated (control) males than to calls from their fathers. High values on component 2 correlated with looking towards the speaker faster, approaching the speaker sooner, and spending more time near the speaker. **B**: Component 2 did not show a significant difference between responses to alarm calls from fathers and unrelated males. High values on component 2 correlated with approaching the speaker sooner and spending more time near the speaker. See Table 2 and Additional file 2 for the quartiles of each behavioral variable and their loadings on the components.

The females paid more attention to advertisement calls from unrelated males than from their fathers, but showed no differences in response to alarm calls from unrelated males and from their fathers (Figure 2, see also Table 2 and Additional file 2, which summarize the data). The components of the females’ responses to advertisement calls accounted for 47%, 15%, and 15% of the variation in the original response behaviors. Component 2, the attention to speaker component, showed that nonestrous females paid more attention to the advertisement calls of the unrelated males than to calls from their fathers (Bonferroni corrected Wilcoxon matched pairs test, Z=−2.395, n=10, P=0.017). High values on component 2 correlated with looking towards the speaker faster, approaching the speaker sooner, spending more time near the speaker, and spending more time in the box area. (After looking towards/approaching the loudspeaker and finding no lemur, sometimes the subject would then approach the nest box and appear to look inside. Because the lemurs are transported from cage to cage using the nest boxes, the nest box may be a second place for the subjects to look for another lemur).

**Table 2:** Quartiles of the behavioral responses to advertisement calls and their loadings on the principal components

**Behavior**	**Quartiles**			**Component loadings**		
	**25**	**50**	**75**	**Component 1**	**Component 2**	**Component 3**
**Latency to leave bottle area**	114.00	214.00	673.00	0.870	0.148	0.051
**Latency to box area**	136.25	376.50	673.00	0.869	0.231	0.127
**Duration in bottle area**	483.00	652.00	813.63	0.841	0.137	0.183
**Latency to speaker area**	697.13	1420.50	1500.00	0.735	−**0**.**585**	−0.101
**Latency to box**	340.00	890.50	1465.88	0.722	0.342	0.487
**Duration look to speaker**	18.25	33.50	61.50	0.628	−0.055	−0.614
**Duration in box**	14.50	221.00	407.75	−0.580	−0.441	−0.367
**Duration in speaker area**	0.00	63.50	218.00	−0.716	**0**.**591**	0.183
**Duration in box area**	115.13	185.75	299.13	−0.513	**0**.**539**	−0.291
**Latency to look to speaker**	22.50	144.00	295.38	−0.266	−**0**.**528**	0.687
**Duration look to box**	0.00	13.75	21.25	−0.518	−0.148	0.480

Components 1, 2, and 3 are 47%, 15%, and 15% percent of the variation, respectively.

Behavioral variables that correlated highly (<−0.5 or >0.5) with component 2 are in bold. Frequency is measured in Herz and time is in frames (resolution of 25 frames/s).

Using component 2 scores, nine of the 10 females paid more attention to the unrelated males’ advertisement calls than to those of their fathers (Figure 2). Components 1 and 3 did not differentiate between responses to fathers’ and control males’ advertisement calls (Bonferroni corrected Wilcoxon Matched Pairs Test, Component 1: Z=−0.561, n=10, p=0.575, Component 3: Z=−1.58, n=10, p=0.114).

The components of the responses to alarm calls accounted for 39%, 22% and 16% of the variation in the original response behaviors. None of the components differentiated between responses to fathers’ and control males’ alarm calls (Figure 2, Bonferroni corrected Wilcoxon Matched Pairs Test, Component 1: Z=−1.172, n=10, P=0.241; Component 2: Z=−0.051, n=10, P=0.959; Component 3: Z=−0.968, n=10, P=0.333).

To exclude the possibility that arousal confounded our results, we measured parameters *most likely* to vary with arousal [43], and tested for differences between the stimulus calls of related and control males. We measured the peak frequency of the fundamental, the call duration, number of modulations and the modulation rate (number of modulations/duration) of the advertisement calls (BatSound Pro 3.31, Pettersson Elektronik AB, Uppsala, Sweden). Peak frequency of the fundamental, call duration, and modulation rate did not differ between the five father-control male dyads (Wilcoxon matched pairs tests, peak frequency of the fundamental: Z=−0.67, n=5, P=0.50; call duration: Z=−1.21, n=5, P=0.23; modulation rate: Z=−1.48, n=5, P=0.14). The number of modulations showed a trend (Wilcoxon matched pairs tests, Z=−1.63, n=5, P=0.10), but was not significantly correlated with the Attention to Speaker component (Spearman Correlation, rho≥−0.099, n=20, P=0.339). Therefore, we concluded that the arousal state of the caller did not confound our results.

## Discussion

We found that male grey mouse lemur advertisement calls, but not alarm calls, contain acoustic patrilineal signatures. Furthermore, females paid more attention to the unrelated males’ advertisement calls than those of their fathers. Though the females were not in estrous at the time, this increased attention to unrelated males suggests that such discrimination may be an important mechanism for inbreeding avoidance.

The two main kin recognition mechanisms proposed for mammals are familiarity and phenotype matching (sensu Widdig [20]: matching an unknown individual either to oneself or to known kin). In our study subject females were equally familiar with the calls of both their fathers and their control males, but this does not exclude the possibility that the females used their own calls and/or calls of their full-siblings as a template against which the stimulus calls were compared [35,44]. (Both males and females give these highly modulated advertisement calls). Thus, inbreeding avoidance could be accomplished if females prefer males with calls that are different from their own and their paternal/full siblings’ calls, and alternatively, kin selection could occur if mouse lemurs give preferential treatment to lemurs with calls similar to their own and their paternal/full siblings’ calls.

Mateo [35] argues that phenotype matching would be selected for in species with (i) a lack of paternal care, (ii) multiple paternity litters, and/or (iii) communal nesting. Thus, the social system of mouse lemurs should favor phenotype matching: (i) Since males do not provide paternal care and do not co-nest or co-forage with their mates or with their young [31,33], this strongly limits the effectiveness of the familiarity-based mechanisms often seen in more gregarious species with cohesive foraging groups (i.e., primates [20,36], elephants [8]). (ii) Mouse lemur litters can have multiple paternities within the same litter [37], thus infant mouse lemurs could be predicted to evolve self-referential phenotype matching to distinguish between full-siblings and maternal half-siblings in the nest. (iii) Given that multiple females may breed in the same nest [31], infant mouse lemurs could potentially encounter paternal half siblings within the other mother’s litter and use self-referential phenotype matching to recognize them. Self-referential phenotype matching has been observed in ground squirrels using olfactory cues [45] and future work on mouse lemurs will aim to distinguish between self-referential phenotype matching and phenotype matching using kin as templates.

The difference in kin recognition between the two call types may be due both to the structure of the call types and to their role in the social system of this nocturnal, solitary foraging mammal. The advertisement call has a highly complex modulated structure that is well-suited to display patrilineal signatures. The alarm call is a shorter, non-frequency modulated call that may provide less opportunity to display the subtle differences between callers that appear necessary for patrilineal signatures. The lack of kinship signatures in the alarm calls also fits well with a prior report of cooperative mobbing of snakes by wild mouse lemurs which resulted in the rescue of an unrelated conspecific [46]. It suggests that mouse lemurs do *not* behave in the wild as if they are using kin signatures from the alarm calls (commonly given during predator mobbing) to selectively give aid to kin [46]. The costs of responding to a related conspecific’s mate advertisement call (inbreeding) may be high enough and the costs of responding to an alarm call low enough, that patrilineal signatures may be more strongly selected for in the advertisement call than the alarm call. Our results on paternal kin recognition, combined with prior work showing differences in maternal kin recognition across call types ([3,47,48]), indicate that the selective pressures that drive the evolution of acoustic kin recognition are not uniform throughout all aspects of the communication system and that kin recognition in different calls may evolve independently.

The costs of sociality for a small-bodied, nocturnal mammal with a dispersed social system may have selected for higher frequencies in the social advertisement calls than in the alarm calls. Alarm calls are typically given in the context of a present threat when crypsis appears to no longer be the primary tactic of predator/threat avoidance [49,50]. In contrast, advertisement calls are social/mating calls and may facilitate interactions in close proximity, leading to an increased risk of detection due to the movements of multiple, rather than one, animal. The increased crypsis offered by the ultrasonic frequencies may help limit eavesdropping opportunities for predatory birds to only movement-related and not vocalization-related acoustic cues [38,39,51]. Additionally, the evolution of patrilineal signatures and kin recognition in these calls may enable listeners to choose *not* to approach the caller, thus avoiding the extra predation risk inherent in approaching the caller should the caller not be an advantageous mate. Such discrimination could be advantageous to both the listener and the caller.

An additional, non-mutually exclusive possibility is that the advertisement call may have been under more selective pressure due to interference from environmental background noise [51]. Radespiel and colleagues [32,52] provide evidence that male mouse lemurs leave their sleeping sites earlier in the night than the females during the breeding season and use that time to go to the females’ sleeping sites and potentially monitor their estrous status. If this early evening/dusk time is critical for finding mates, it may coincide with a time of heightened background noises, including rising winds due to changing temperatures and increased insect activity (S. Kessler, pers. obs., 2010). This increased noise at this time of night could select for the calls to be given at higher frequencies, thus enabling individuals to maintain a better signal-to-noise ratio if there is a lot of background noise in the lower frequencies [51] . In addition, in this context, where the caller and receiver are in close proximity (female inside the sleeping site, and male outside) it may be advantageous that the ultrasonic frequencies will rapidly scatter and not be heard by other conspecific competitors [51].

This suggests that high predation pressure and basal mammalian traits such as small body size and dispersed social systems select for paternal kin recognition in the high frequency and ultrasonic range, thus limiting the risks of inbreeding and being eavesdropped by predators or competitor conspecifics. Future analyses will determine which acoustic parameters make this kin recognition possible and will involve experimentally manipulating the acoustic parameters.

To our knowledge, our study is the first to demonstrate that that acoustic paternal kin recognition in mammals can evolve independently of a large brain, cohesive foraging groups, and a complex social system, and that it can also evolve in small-bodied, nocturnal solitary foragers whose main predator defense is crypsis. Given that more complex forms of sociality with cohesive foraging groups are thought to have evolved from an ancestral solitary forager much like the grey mouse lemur [29,53], this suggests that mechanisms for kin recognition like those seen in this solitary forager may have been the foundation from which more complex forms of kin-based sociality evolved.

## Conclusions

We provide the first evidence for paternal kin recognition using vocalizations in a small-brained, nocturnal, solitary foraging mammal, indicating that high predation, and basal mammalian traits, such as small body size and a dispersed social system, may select specifically for paternal kin recognition in the high frequency/ultrasonic ranges, thus limiting the risks of inbreeding and eavesdropping by predators or competitor conspecifics. Paternal kin recognition via vocalizations in mammals is not dependent upon a large brain and high social complexity, but may already have been an integral part of the dispersed social networks from which more complex, kin-based sociality is thought to have evolved.

## Methods

### Patriline signatures

All calls used for this study were from the sound archive of the Institute of Zoology, University of Veterinary Medicine Hannover or newly recorded in 2008. All recordings were made with one of two previously published methods. For the first we connected the high frequency output of a bat detector (U30, Ultra Sound Advice, frequency range: >100 kHz) via a control filter unit (Pettersson box F2000) to a high-speed A/D card (DAS 16/330) in a laptop (Compaq Armada) equipped with the recording software BatSound Pro 3.31 (Pettersson Elektronik AB, Uppsala, Sweden). For additional details see Scheumann and colleagues [54]. For the second set-up consisted of connecting the high frequency output of a bat detector (frequency range: 8–100 kHz) to a high-speed analog-to-digital (A/D) card in a laptop (sampling frequency: 200–500 kHz) using the program NiDisk (for more details see Leliveld and colleagues [40]). All calls were recorded at 16-bit per sample with a sampling frequency of 200 kHz or higher, and when higher, were resampled to 200 kHz. Mating calls were recorded during the breeding season from the male in the presence of a female. Alarm calls were recorded in disturbance/social contexts (novel object in the cage, after hearing a novel sound, predator call, conspecific alarm call, or in the context of a social interaction). The work in this study was licensed by the Bezirksregierung Hannover, Germany (reference number: 509.6-42502-03/660) and the Arizona State University Institutional Animal Care and Use Committee (protocol 08-966R, 1/31/2008). All research complied with the animal care guidelines and the applicable national laws in Germany and the United States.

We analyzed advertisement and alarm calls from three patrilines housed at the University of Veterinary Medicine Hannover. Matrilineal and patrilineal relatedness values were calculated for all dyads within and between patrilines (see Tables 3 and 4) using breeding colony records maintained since the founding of the colony in 1985 and containing a pedigree depth of up to nine generations. When a dyad had a common ancestor who was a maternal relative for one individual and a paternal relative for the other, that ancestor’s portion of the relatedness value was divided by two and half was attributed to the dyad’s maternal relatedness and half to the dyad’s paternal relatedness. The paternity of one male (not a stimulus male) within the pedigree was both unknown and could have influenced calculations. This case was resolved with the goal of maximizing inbreeding, thus minimizing genetic separation between patrilines and being conservative regarding our hypotheses. Mean patrilineal relatedness within and between patrilines was 0.426 and 0.073, respectively (Table 3). Mean matrilineal relatedness within and between patrilines was 0.041 and 0.053, respectively (Table 4). When animals have r values higher than 0.5, they are slightly inbred. (For colony management details: [55]).

**Table 3:** Patrilineal relatedness within and between the patrilines in the patriline signature analysis

	**Eddie**	**Beetle**	**Amigo**	**Adrian**	**Xaver**	**Uli**	**Yves**	**Vito**	**Vincent**
**Eddie**									
**Beetle**	**0**.**500**								
**Amigo**	**0**.**281**	**0**.**516**							
**Adrian**	0.000	0.000	0.000						
**Xaver**	0.043	0.027	0.021	**0**.**500**					
**Uli**	0.111	0.098	0.056	**0**.**250**	**0**.**500**				
**Yves**	0.195	0.141	0.100	0.063	0.094	0.117			
**Vito**	0.113	0.086	0.058	0.063	0.063	0.070	**0**.**514**		
**Vincent**	0.113	0.086	0.058	0.063	0.063	0.070	**0**.**514**	**0**.**257**	

Relatedness within the three patrilines is shown in bold.

**Table 4:** Matrilineal relatedness within and between the patrilines in the patriline signature analysis

	**Eddie**	**Beetle**	**Amigo**	**Adrian**	**Xaver**	**Uli**	**Yves**	**Vito**	**Vincent**
**Eddie**									
**Beetle**	**0**.**000**								
**Amigo**	**0**.**031**	**0**.**016**							
**Adrian**	0.000	0.000	0.000						
**Xaver**	0.066	0.059	0.035	**0**.**000**					
**Uli**	0.193	0.070	0.066	**0**.**000**	**0**.**039**				
**Yves**	0.023	0.031	0.014	0.063	0.094	0.055			
**Vito**	0.059	0.031	0.140	0.000	0.041	0.054	**0**.**014**		
**Vincent**	0.059	0.031	0.140	0.000	0.041	0.054	**0**.**014**	**0**.**257**	

Relatedness within the three patrilines is shown in bold.

**Figure 3:**
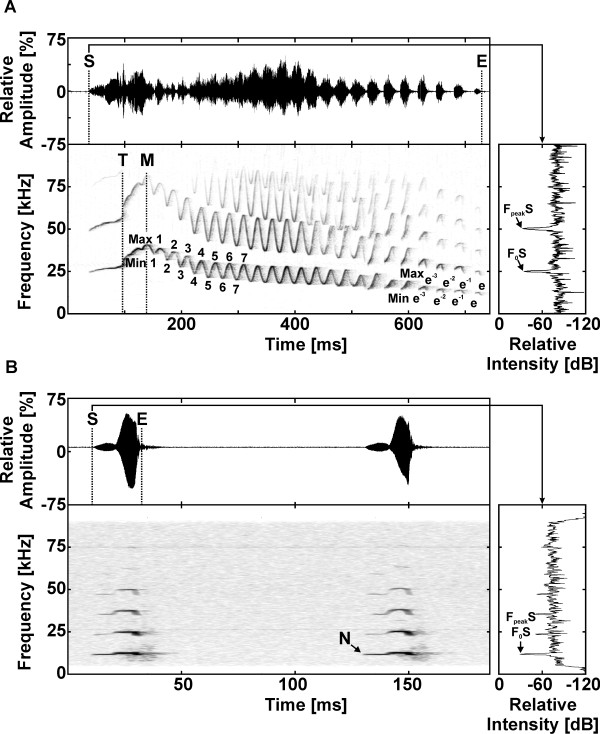
**Oscillogram**, **spectrogram**, **and power spectrum showing the highly modulated advertisement call** (**A**) **and the almost nonfrequency modulated alarm call.** (**B**) Some acoustic parameters are depicted. F_peak_S is the peak frequency of the start and F_0_S is the fundamental frequency of the start. Figure produced in BatSound Pro 3.31 (Pettersson Elektronik AB, Uppsala, Sweden) according to Leliveld and colleagues [40]. See Additional files 3 and 4 for more information.

We measured ten advertisement calls and ten alarm call series from each of nine adult males, three males/patriline. Male ages in years when advertisement calls were recorded are: patriline 1: 4–9 (mean=6), patriline 2: 2–5 (mean=3), patriline 3: 4–6 (mean=5). Male ages in years when alarm calls were recorded are: patriline 1: 4–6 (mean=5), patriline 2: 3–6 (mean 5), patriline 3: 2–4 (mean=3). All males were sexually mature at the time of recording. (Mouse lemurs are sexually mature at one year old [56]). We used the same macros as Leliveld and colleagues [40] in Signal 4.0 (Engineering Design, Belmont, USA). See Figure 3 for sample oscillograms, spectrograms, and power spectrums showing how measurements were made and Additional files 3 and 4 for definitions of advertisement call and alarm call parameters, respectively. Figure 3, Additional file 3 and Additional file 4 were produced according to Leliveld and colleagues [40]. We measured 45 acoustic parameters in the advertisement calls and 10 parameters in the alarm calls. These parameters were chosen to provide a detailed characterization of the contour of the fundamental frequency for each call type. As is evident in Figure 3, the advertisement call is far more structurally complex than the alarm call, thus more parameters are required to characterize it.

We used principal components analysis (Factor analysis, principal components method on the correlation matrix, no rotation, SPSS 20, Chicago, USA) to reduce the data to two components for each call type. Then, for each call type, the component scores were put into a permutated linear discriminant function analysis with individual nested within patriline [57]. Cross-validation was performed with the leave-one-out method (Mundry, R. pers. com. 2008). Alpha was set at 0.05. This statistical technique of first conducting principal components analysis for parameter reduction [42] and then putting the component scores into a discriminant function analysis is widely accepted in the acoustic literature across taxa (i.e., gibbons [58], langurs [59], wolves [60], baboons [3], macaques [47], mouse lemurs [40], flycatchers [61], bats [62,63]). Such parameter reduction is important because the permutated discriminant function analysis is sensitive to the number of predictor variables [57] and the principal components analysis enables one to retain more information from the original parameters than could be included when just a small subset of the original parameters was chosen [42].

We verified that the patrilineal signatures we found are related to patrilineal relatedness, not matrilineal relatedness between subjects by performing Mantel tests investigating the relationship between 1) acoustic dissimilarity and paternal relatedness and 2) acoustic dissimilarity and maternal relatedness. Paternal and maternal relatedness values are shown in Tables 3 and 4. For both tests acoustic dissimilarity was calculated as follows using an acoustic dissimilarity index (Kastein HB, Winter R, Vinoth Kumar AK, Sripathi K, Schmidt S: Perception of individuality in bat vocal communication: discrimination between, or recognition of, interaction partners?, unpublished).

First each call parameter for each call was normalized to have a value between 0 and 1 using: *p*_*ni*_ = (*p*_*i*_ - *p*_*min*_)/(*p*_*max*_ - *p*_*min*_) where p_ni_ is the normalized parameter value, p_i_ is the raw parameter value, and p_max_ and p_min_ are the maximum and minimum values of that parameter across the entire dataset. Second, we calculated a dissimilarity index for each parameter for each individual:

${\text{Dissimiliarity}}_{\text{parameter per individual}}=\sqrt{\frac{\sum_{i=1}^{n_{c}}{\left(p_{ni}-p_{median}\right)}^{2}}{n_{c}}}$P_ni_ is the normalized parameter calculated in the previous formula, p_median_ is the median for that parameter across the whole dataset, and n_c_ is the number of calls per individual. Third, we combined these dissimilarity indexes across parameters within individuals using root mean squares. We followed the parameter groupings of the principal components analysis. Thus we calculated, for each individual, a root mean square of the acoustic parameters in component 1, and a second root mean square of the acoustic parameters in component 2. Fourth, we used these two dissimilarity indexes to calculate Euclidian distances between all possible dyads, producing a matrix of acoustic dissimilarity. We transformed the relatedness matrices (Tables 3 and 4) into relatedness *dis*similarity matrices by subtracting each value from 1 (a father-son dyad is related patrilineally by 0.5, thus they would also have a patrilineal genetic *dis*similarity index of 0.5). We then conducted Mantel tests in Mantel 2.0 [64] using 1000 permutations to test for a correlation between acoustic dissimilarity and patrilineal genetic dissimilarity and between acoustic dissimilarity and matrilineal genetic dissimilarity.

### Patriline recognition

We conducted playback experiments at the University of Veterinary Medicine Hannover in 2008. Ten adult *nonestrous* females (ages 2–8 years) heard advertisement calls and alarm calls from their genetic father and an unrelated control male (r≤0.141) played in a randomized order. As can be seen in Tables 5 and 6, patrilineal relatedness between fathers and daughters was high (mean=0.506) while matrilineal relatedness was low (mean=0.019). In contrast, both patrilineal relatedness and matrilineal relatedness was low between the females and their control males (mean patrilineal relatedness: 0.054, mean matrilineal relatedness: 0.049). Thus we do not expect matrilineal relatedness to have been confounded with patrilineal relatedness. Advertisement calls were recorded from fathers aged 2–8 years (mean=6) and from control males aged 2–9 years (mean=7) at the time of recording. Alarm calls were recorded from fathers aged 5–8 years (mean=6) and from control males aged 6–8 years (mean=8). Mouse lemurs are sexually mature at one year old [56], thus all calls were recorded from adult males. Additionally, because Leliveld and colleagues [65] found that mouse lemurs did not respond differently to calls from lemurs of different ages, we do not expect age to have confounded our results. We used calls from a total of seven males, from which five were fathers and four were unrelated males. Some fathers were also used as unrelated males for other females. Familiarity was controlled in that each female had been housed in the same room as her father and her control male for longer than six months including time during the breeding season when mating calls and alarm calls are frequently heard in the animal rooms. Lemurs in the colony have visual, olfactory, and auditory contact with the other lemurs in their rooms. Three father-daughter dyads and three control-male–female dyads had a few hours of interaction with each other. For two females (one litter: Vicky and Vivian) the father was not removed from the mother’s cage until a few hours after the birth was discovered. (Normally the father is removed from the mother’s cage several days before birth and is never housed in the same cage as his daughters. Adults are typically caged with 1–3 other adults, and if that is not possible, they are caged alone until a cage-mate is available). Additionally one other father-daughter dyad (Yeti-Tipi) and three control male–female dyads had a few hours of contact with each other when they were briefly put together in the recording chamber when recordings were made for this study or previous studies. Therefore, the number of father-daughter dyads and control male–female dyads that had prior experience with each other was equal and thus balanced. For each of these three father-daughter dyads and three control male–female dyads the maximum total time that they would have had together was a few hours, thus we do not expect this to have influenced the playback results and consider the females be equally familiar with both their fathers and their control males because they have shared a room with both males for more than 6 months and not been in the same cage for more than a few hours. During this study no female heard recordings that were made during a recording session in which she participated. Four control male–female dyads and one father daughter dyads were currently sharing a room at the time of the experiments. It was not possible to standardize when in the females’ lives or for how long they shared the room with their fathers and control males because, over the course of their lives, the housing arrangements had always been dependent upon the needs of on-going experiments and the breeding program. We chose the subjects we did to maximize sample size and standardize familiarity as much as possible, given the housing histories and relatedness constraints within the colony. Additional file 5 provides the details of how familiar each female was with her father and her control male.

**Table 5:** **Patrilineal relatedness between the female**-**father dyads and between female**-**control male dyads**

**Female**	**Father**	**Relatedness**	**Control**	**Relatedness**
**Tasha**	Xaver	0.516	Emil	0.035
**Tipi**	Yeti	0.517	Zambo	0.076
**Tweety**	Xaver	0.516	Emil	0.035
**Undine**	Zambo	0.508	Xaver	0.032
**Vicky**	Beetle	0.500	Adam	0.063
**Vivian**	Beetle	0.500	Adam	0.063
**Zizi**	Adrian	0.500	Zambo	0.055
**Zoly**	Adrian	0.500	Zambo	0.055
**Zuby**	Adrian	0.500	Zambo	0.055
**Zwipsy**	Adrian	0.500	Zambo	0.070

**Table 6:** **Matrilineal relatedness between the female**-**father dyads and between the female**-**control male dyads**

**Female**	**Father**	**Relatedness**	**Control**	**Relatedness**
**Tasha**	Xaver	0.037	Emil	0.016
**Tipi**	Yeti	0.048	Zambo	0.043
**Tweety**	Xaver	0.037	Emil	0.016
**Undine**	Zambo	0.070	Xaver	0.053
**Vicky**	Beetle	0.000	Adam	0.063
**Vivian**	Beetle	0.000	Adam	0.063
**Zizi**	Adrian	0.000	Zambo	0.055
**Zoly**	Adrian	0.000	Zambo	0.055
**Zuby**	Adrian	0.000	Zambo	0.055
**Zwipsy**	Adrian	0.000	Zambo	0.070

Subjects were habituated to the sound attenuated testing chamber though previous experiments and an extra 30 min. habituation session prior to the first session where a stimulus was presented. Each female participated in six testing sessions. Within each session the female heard four stimulus types: a mate advertisement call from her father, a mate advertisement call from her control male, an alarm call series from her father and an alarm call series from her control male. Each female heard novel call exemplars from the same pair of males in each of the six sessions (except for two females, Tasha and Tweety, from whose father only two advertisement and alarm call sequences could be obtained). Within a session, the stimulus types were played in a randomized order and separated by a minimum rehabituation time of five minutes (previously shown to be an adequate rehabituation time for mouse lemurs [66]). Sessions were conducted within the first three hours of the subjects’ active period (dark period of the light cycle). Each session lasted between approximately 30 and 90 min. Subjects participated in only one session per day with a minimum of one day and a maximum of six weeks between sessions. All females’ scores for further analyses were medians calculated across the sessions per stimulus type for each behavioral variable.

**Figure 4:**
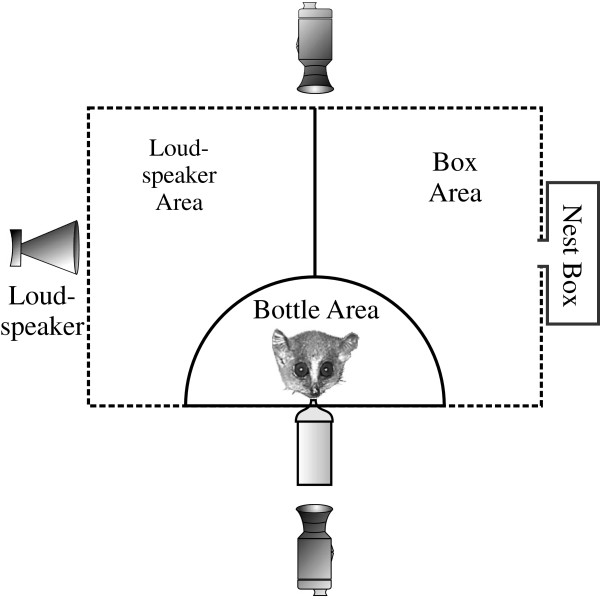
**Cage set**-**up for playback experiments**. **The close camera is behind the drinking bottle and the wide**-**angle camera is behind the lemur.** Latency to look to the speaker, duration of the look to the speaker, and duration of looking to the box were coded on the close camera. Latency to speaker area, duration in loudspeaker area, duration in bottle area, latency to box area, latency to box, latency to leave bottle area, duration in box area, duration in box were coded on the wide-angle camera. The sound attenuated chamber was 225 cm by 340 cm by 225 cm. The cage was 80 cm by 50 cm by 87 cm. See Additional file 6 for an ethogram of the scored behavioral variables.

Each stimulus consisted of one advertisement call (typically 500–600 ms) or an alarm calls series of equal length to the advertisement call of that male (typically 5–8 calls). This stimulus was repeated three times, separated by about 3.6 seconds (mean intercall interval between advertisement calls given by wild mouse lemurs [66]). Total stimulus length was approximately 12 sec. Stimuli were filtered in BatSound Pro 3.31 (low pass: 80 kHz, high pass 5 kHz), prepared in Signal 4.0., and played at 75 ±1 dB at a distance of 80 cm (RMS measurement, Brüel und Kjær Measuring Amplifier Type 2610) while the lemur licked juice from a bottle in a sound-attenuated chamber. The juice bottle guaranteed that the distance between the loudspeaker and the lemur’s head was the same across all stimuli presentations, for all sessions, for all subjects. For cage set-up see Figure 4, and for additional technical details of playbacks and video analysis see Scheumann and Zimmermann [66]. We observed the subjects’ behavior from outside the chamber on the camcorder’s display screen to avoid influencing the subject. We conducted a frame-by-frame analysis during one min. after the onset of the playback in Interact 8.0.4. (Mangold, Arnstorf, Germany) analyzing 11 behavioral variables. See Additional file 6 for behavioral ethogram. Videos were muted and assigned random numbers before scoring, thus, as it was impossible to identify individuals on video, the experimenter was blind, while coding, to both the lemur’s identity and to what stimulus was played. When the behavioral measures for the first and last sessions were compared, no habituation effects were found (Wilcoxon matched pairs tests on each of the four stimulus types, P>0.05). Intra-observer reliability was confirmed by reanalyzing 20 videos (17%); each pair of observations for each variable were not significantly different (Bonferroni corrected paired T-test, test-wide alpha>0.05) and were significantly correlated (Bonferroni corrected Spearman correlation, rho≥0.73, test-wide alpha<0.05).We ran principal components analysis on the behavioral data of the advertisement calls and the alarm calls (advertisement calls: PROC FACTOR, method=principal, SAS, Cary, USA; alarm calls: Factor analysis, principal components method, SPSS 20).We used a principal components analysis because it enabled us to simultaneously consider several behavioral responses which were coded as separate variables but are different measurements of the same underlying “latent” variable [42]. This is important because not all of the animals show the same behavioral responses. For example, one might run into the speaker area while another might look towards the speaker but not go over to it. Both demonstrate heightened attention to the speaker, and thus are considered measurements of the underlying latent variable ‘Attention to Speaker.’

For each call type we analyzed the 11 behavioral variables and obtained a set of three principal components. These components explained 77% and 77% of the variation in the behavioral responses to advertisement calls and alarm calls, respectively. We conducted Bonferroni corrected Wilcoxon matched pairs tests on the component scores for each component, testing for differences between responses to fathers and unrelated males for each call type (SPSS 20, Chicago, USA). Test-wide alpha per call type was set at 0.05.

## Competing interests

The authors declare that they have no competing interests.

## Authors' contributions

SEK initiated, participated in designing, and conducted the study, ran the statistical tests, and wrote the manuscript. MS, LTN, and EZ contributed to the design and development of the ideas within the study, provided mentoring, and participated in the preparation of the manuscript. All authors have read and approved this manuscript.

## Supplementary Material

Additional file 1 **Quartiles of the acoustic parameters measured from the alarm calls and their loadings on the principal components.** Frequency is measured in Herz and time in milliseconds. Click here for file

Additional file 2 **Quartiles of the behavioral responses to advertisement calls and their loadings on the principal components.** Frequency is measured in Herz and time is in frames (resolution of 25 frames/s). Click here for file

Additional file 3 **Definitions and formulas of the acoustic parameters measured**/**calculated from the advertisement calls for the patriline signature analysis.** Provides more information on how the advertisement calls were measured. Click here for file

Additional file 4 **Definitions and formulas of the acoustic parameters measured**/**calculated from the alarm calls for the patriline signature analysis.** Provides more information on how the alarm calls were measured. Click here for file

Additional file 5 **Housing histories of the female**-**father and female**-**control male dyads.** This table provides the details on how familiar the females were with their fathers and with their control males before the playback experiments. Click here for file

Additional file 6 **Ethogram for video analysis.** This table defines the behavioral variables measured in the video analyses. Click here for file
