# Supplementary material for: Paternal kin recognition in the high frequency / ultrasonic range in a solitary foraging mammal
Source: BMC Ecol. 2012 Nov 30;12:26. doi: 10.1186/1472-6785-12-26 (PMC3537692; doi:10.1186/1472-6785-12-26)
Supplement: Additional file 6 — Ethogram for video analysis. This table defines the behavioral variables measured in the video analyses. [file 1472-6785-12-26-S6.doc]

**Ethogram for Video Analysis**

| **Name** | **Camera** | **Definition** |
| --- | --- | --- |
| Duration look to speaker | Close | **Start:** Head has been turned 45 degrees to the loudspeaker.  **End:** Head has been turned 45 degrees away from the loudspeaker. |
| Duration look to box | Close | **Start:** Head has been turned 45 degrees to the box.  **End:** Head has been turned 45 degrees away from the box. |
| Latency to look to loudspeaker | Close | **Start:** First playback starts.  **End:** Head is turned 45 degrees towards the loudspeaker. |
| Duration in loudspeaker area | Wide | **Start:** Front half of lemur’s body is over the line into speaker area.  **End:** Front half of lemur’s body is over the line into another area. |
| Duration in box area | Wide | **Start:** Front half of lemur’s body is over the line into box area.  **End:** Front half of lemur’s body is over the line into another area. |
| Duration in box | Wide | **Start:** Head is fully in the box.  **End:** Front half of lemur’s body is outside the box. |
| Latency to speaker area | Wide | **Start:** First playback starts.  **End:** Front half of lemur’s body is over the line into the speaker area |
| Latency to box area | Wide | **Start:** First playback starts.  **End:** Front half of lemur’s body is over the line into the box area. |
| Latency to box | Wide | **Start:** First playback starts.  **End:** Head is fully in the box. |
| Duration in bottle area | Wide | **Start:** When the first playback starts or lemur re-enters bottle area by crossing the line with the front half of her body.  **End:** Front half of lemur’s body is over the line into another area. |
| Latency to leave bottle area | Wide | **Start:** First playback starts.  **End:** Front half of lemur’s body is over the line into another area. |
